# Blood Pressure Variability and Cardiovascular Risk in the PROspective Study of Pravastatin in the Elderly at Risk (PROSPER)

**DOI:** 10.1371/journal.pone.0052438

**Published:** 2012-12-20

**Authors:** Rosalinde K. E. Poortvliet, Ian Ford, Suzanne M. Lloyd, Naveed Sattar, Simon P. Mooijaart, Anton J. M. de Craen, Rudi G. J. Westendorp, J. Wouter Jukema, Christopher J. Packard, Jacobijn Gussekloo, Wouter de Ruijter, David J. Stott

**Affiliations:** 1 Department of Public Health and Primary Care, Leiden University Medical Center, Leiden, The Netherlands; 2 Robertson Centre for Biostatistics, University of Glasgow, Glasgow, United Kingdom; 3 Department of Vascular Biochemistry, University of Glasgow, Glasgow, United Kingdom; 4 Department of Gerontology and Geriatrics, Leiden University Medical Center, Leiden, The Netherlands; 5 Institute for Evidence-Based Medicine in Old Age, Leiden, The Netherlands; 6 Netherlands Consortium for Healthy Ageing, Leiden, The Netherlands; 7 Department of Cardiology, Leiden University Medical Center, Leiden, The Netherlands; 8 Academic Section of Geriatric Medicine, University of Glasgow-Faculty of Medicine, Glasgow Royal Infirmary, Glasgow, United Kingdom; University of Oxford, United Kingdom

## Abstract

Variability in blood pressure predicts cardiovascular disease in young- and middle-aged subjects, but relevant data for older individuals are sparse. We analysed data from the PROspective Study of Pravastatin in the Elderly at Risk (PROSPER) study of 5804 participants aged 70–82 years with a history of, or risk factors for cardiovascular disease. Visit-to-visit variability in blood pressure (standard deviation) was determined using a minimum of five measurements over 1 year; an inception cohort of 4819 subjects had subsequent in-trial 3 years follow-up; longer-term follow-up (mean 7.1 years) was available for 1808 subjects. Higher systolic blood pressure variability independently predicted long-term follow-up vascular and total mortality (hazard ratio per 5 mmHg increase in standard deviation of systolic blood pressure = 1.2, 95% confidence interval 1.1–1.4; hazard ratio 1.1, 95% confidence interval 1.1–1.2, respectively). Variability in diastolic blood pressure associated with increased risk for coronary events (hazard ratio 1.5, 95% confidence interval 1.2–1.8 for each 5 mmHg increase), heart failure hospitalisation (hazard ratio 1.4, 95% confidence interval 1.1–1.8) and vascular (hazard ratio 1.4, 95% confidence interval 1.1–1.7) and total mortality (hazard ratio 1.3, 95% confidence interval 1.1–1.5), all in long-term follow-up. Pulse pressure variability was associated with increased stroke risk (hazard ratio 1.2, 95% confidence interval 1.0–1.4 for each 5 mmHg increase), vascular mortality (hazard ratio 1.2, 95% confidence interval 1.0–1.3) and total mortality (hazard ratio 1.1, 95% confidence interval 1.0–1.2), all in long-term follow-up. All associations were independent of respective mean blood pressure levels, age, gender, in-trial treatment group (pravastatin or placebo) and prior vascular disease and cardiovascular disease risk factors. Our observations suggest variability in diastolic blood pressure is more strongly associated with vascular or total mortality than is systolic pressure variability in older high-risk subjects.

## Introduction

In daily practice and all major clinical guidelines [1]–[5], ‘usual’ or average blood pressure is considered to be the key or most important measure determining risk of cardiovascular disease (CVD); reductions in average blood pressure are generally thought to account for the benefits of antihypertensive drugs [1]–[9]. However, recently Rothwell *et al.* [10] has questioned the usual blood-pressure hypothesis, suggesting that visit-to-visit variability in blood pressure (assessed across multiple visits) may have an important additional role in increasing risk of vascular events, and in particular stroke. Visit-to-visit variability in blood pressure is increased in cohorts at high risk of stroke [11], [12]. A secondary analysis of several randomised controlled trials found that visit-to-visit variability in systolic blood pressure and episodic hypertension were strong predictors of stroke, independent of mean systolic blood pressure [13]. In addition the adverse effects of variable blood pressure may stretch beyond stroke. In a population-based study of US adults, higher levels of visit-to-visit variability in systolic blood pressure were associated with increased all-cause mortality [14].

However the risks associated with visit-to-visit variability of blood pressure in older age are less clear; some investigators have suggested such associations with visit-to-variability in systolic blood pressure may decrease with advancing age [13]. Therefore, we aimed to establish whether visit-to-visit variability in blood pressure in older patients is associated with increased risk of incident CVD. We performed an analysis of the PROspective Study of Pravastatin in the Elderly at Risk (PROSPER) cohort [15].

**Figure 1:**
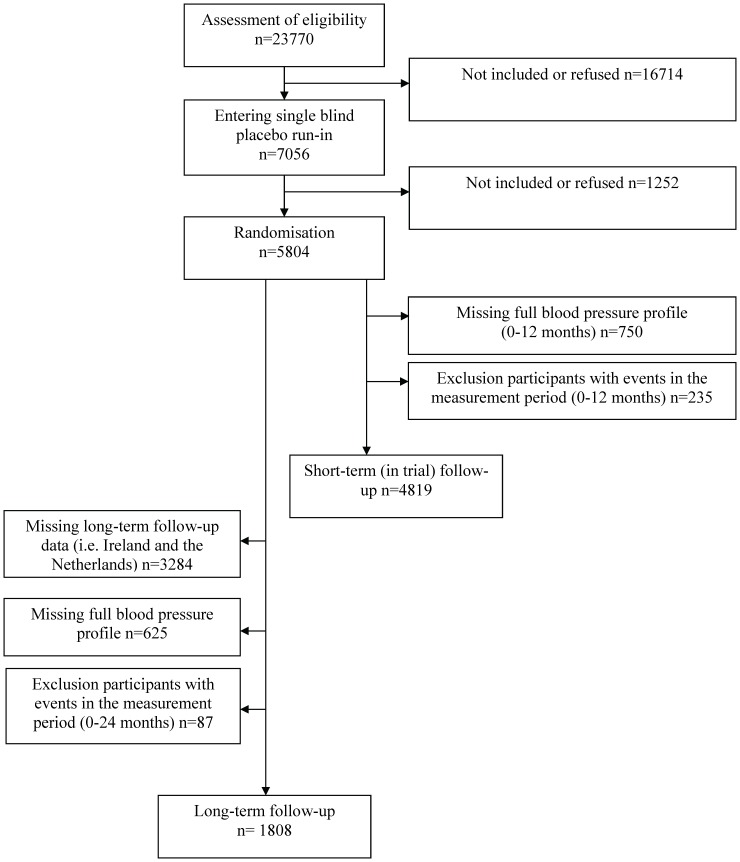
Flow chart.

## Methods

### Study Design

Details of the design and outcome of PROSPER have been published elsewhere [15]–[17]. Between December 1997 and May 1999 a total of 5804 individuals were screened and enrolled in Scotland, Ireland and the Netherlands. Men and women aged 70–82 years were recruited if they had either pre-existing vascular disease (coronary, cerebral, or peripheral) or raised risk of such disease because of smoking, hypertension or diabetes. Plasma total cholesterol was required to be 4.0–9.0 mmol/L and triglyceride concentrations ≤6.0 mmol/L. Individuals with poor cognitive function (Mini-Mental State Examination score <24 points) were excluded. The level of blood pressure was not part of the inclusion or exclusion criteria. The institutional ethics review boards of all centres approved the protocol and all participants gave written informed consent. The protocol adhered to the principles of the Declaration of Helsinki.

**Table 1:** Baseline characteristics of participants with a short-term and long-term follow-up.

	Follow-up	
	Short-term (whole cohort) (n = 4819)	Long-term (Scottish sub-cohort) (n = 1808)
**Continuous variates (mean, SD)**		
Age (years)	75.2 (3.3)	75.2 (3.4)
Systolic blood pressure (mmHg)	154.7 (21.6)	153.7 (20.8)
Diastolic blood pressure (mmHg)	83.9 (11.4)	82.9 (10.8)
Pulse pressure (mmHg)	70.8 (18.1)	70.9 (17.7)
Body mass index (kg/m^2^)	26.9 (4.2)	26.8 (4.1)
Alcohol (units per week)*	5.3 (9.4)	4.6 (8.2)
Total cholesterol (mmol/L)	5.7 (0.9)	5.7 (1.0)
Low-density lipoprotein cholesterol (mmol/L)	3.8 (0.8)	3.8 (0.8)
High-density lipoprotein cholesterol (mmol/L)	1.3 (0.4)	1.3 (0.4)
Triglycerides (mmol/L)	1.5 (0.7)	1.6 (0.7)
Mini-Mental State Examination (pts)	28.1 (1.5)	28.3 (1.4)
Barthel index (pts)	13.7 (1.0)	13.8 (0.8)
Instrumental activities of daily-living (pts)	19.8 (0.7)	19.8 (0.6)
**Categorical variates (n, %)**		
Men	2339 (48.5)	876 (48.5)
Current smoker	1262 (26.2)	472 (26.1)
History of diabetes mellitus	492 (10.2)	144 (8.0)
History of hypertension	3016 (62.6)	1077 (59.6)
History of cardiovascular disease†	2086 (43.3)	865 (47.8)

SD Standard deviation.

* 1 unit = 60 ml distilled spirits, 170 ml wine or 300 ml beer.

† Any of stable angina, intermittent claudication, stroke, transient ischemic attack, myocardial infarction, peripheral arterial disease surgery, or amputation for vascular disease more than 6 months before study entry.

### Blood Pressure Measurements

Sitting blood pressure was measured once at baseline and at follow-up visits every three months during the randomised phase of the trial (mean follow-up 3.2 years) with a fully automatic electronic sphygmomanometer (Omron M4®) by trained research nurses.

### Outcomes and Follow-up

The outcomes for this study were incidence of cardiovascular events, including definite or suspected death from coronary heart disease or non-fatal myocardial infarction (CHD/MI), fatal or non-fatal stroke, heart failure hospitalisation, vascular mortality and total mortality.

**Table 2:** Hazard Ratio’s for the endpoints associated with quartiles of the standard deviation (SD) of systolic blood pressure.

	Short-term follow-up (n = 4819)				
	Quartile of SD of systolic blood pressure, range in mmHg				
Outcomes	Group 1 (n = 1139) ≤9	Group 2 (n = 1194) >9–12.5	Group 3 (n = 1266) >12.5−≤17	Group 4 (n = 1220) >17	P for heterogeneity
Coronary events (n = 407)	1 (ref)	0.8 (0.6–1.1)	1.0 (0.7–1.3)	1.0 (0.8–1.3)	0.563
Fatal/non-fatal stroke (n = 158)	1 (ref)	1.0 (0.6–1.6)	1.1 (0.7–1.8)	1.2 (0.8–1.9)	0.764
Heart failure hospitalisation (n = 144)	1 (ref)	0.5 (0.3–0.8)	0.8 (0.5–1.3)	1.0 (0.7–1.5)	0.019
Vascular mortality (n = 172)	1 (ref)	0.8 (0.5–1.2)	1.0 (0.6–1.5)	0.9 (0.6–1.3)	0.741
Total mortality (n = 330)	1 (ref)	1.0 (0.7–1.3)	1.1 (0.8–1.6)	1.0 (0.8–1.4)	0.676
	**Long-term follow-up (n = 1808)**				
	**Quartile of SD of systolic blood pressure, range in mmHg**				
	**Group 1 (n = 412) ≤10.5**	**Group 2 (n = 428) >10.5−≤13**	**Group 3 (n = 471) >13−≤16.5**	**Group 4 (n = 497) >16.5**	**P for heterogeneity**
Coronary events (n = 248)	1 (ref)	0.9 (0.6–1.3)	1.3 (0.9–1.9)	1.2 (0.8–1.7)	0.155
Fatal/non-fatal stroke (n = 245)	1 (ref)	1.0 (0.7–1.5)	1.3 (0.9–2.0)	1.3 (0.9–1.8)	0.329
Heart failure hospitalisation (n = 216)	1 (ref)	0.8 (0.5–1.3)	1.4 (1.0–2.1)	1.2 (0.8–1.8)	0.044
Vascular mortality (n = 315)	1 (ref)	1.1 (0.7–1.5)	1.5 (1.0–2.1)	1.6 (1.1–2.2)	0.016
Total mortality (n = 735)	1 (ref)	1.2 (1.0–1.5)	1.4 (1.1–1.7)	1.5 (1.2–1.8)	0.006

Data are presented as Hazard Ratios and 95% Confidence Intervals.

Adjustment for randomized treatment, country (short-term follow-up only), mean systolic blood pressure, age, gender, current smoker, histories of diabetes, hypertension, cardiovascular disease, cerebrovascular disease & peripheral vascular disease, body mass index, high-density lipoprotein and low-density lipoprotein.

All in-trial endpoints were assessed by the PROSPER Endpoints Committee, which was blinded to study medication. For this study the in-trial outcomes occurring over a maximum of 3 years (mean 2.3 years), following *one* year of blood pressure observations (i.e. five blood pressure measurements) were analysed. This follow-up was considered ‘short-term’.

Routine health data on morbidity and mortality for the Scottish sub-group (including post-trial follow-up) were obtained from the Information Services Division, a division of National Services Scotland, part of ***National Health Service*** Scotland. The data obtained included the Scottish Morbidity Records (SMR) - SMR00 outpatient attendances; SMR01 general acute inpatient and day case discharges; SMR04 psychiatric admissions, residents and discharges; SMR06 cancer registrations, and General ***Register*** Office for Scotland death registrations. The outcomes for the Scottish sub-group were followed up over a maximum of 9.3 years (mean 7.1), following *two* years of blood pressure observations (with nine blood pressure measurements). This was considered the ‘long-term follow-up’.

**Table 3:** Hazard Ratio’s for the endpoints associated with quartiles of the standard deviation (SD) of diastolic blood pressure.

	Short-term follow-up (n = 4819)				
	Quartile of SD of diastolic blood pressure, range in mmHg				
Outcomes	Group 1(n = 1127) ≤4.8	Group 2 (n = 1157) >4.8−≤6.5	Group 3(n = 1319)>6.5−≤9	Group 4(n = 1216) >9	P for heterogeneity
Coronary events (n = 407)	1 (ref)	1.0 (0.8–1.4)	1.0 (0.8–1.4)	1.3 (1.0–1.8)	0.096
Fatal/non-fatal stroke (n = 158)	1 (ref)	0.8 (0.5–1.2)	1.0 (0.6–1.5)	0.6 (0.4–1.0)	0.211
Heart failure hospitalisation (n = 144)	1 (ref)	1.1 (0.7–1.9)	1.1 (0.7–1.9)	1.8 (1.1–3.0)	0.027
Vascular mortality (n = 172)	1 (ref)	0.8 (0.5–1.2)	1.3 (0.8–1.9)	1.3 (0.9–2.1)	0.056
Total mortality (n = 330)	1 (ref)	0.7 (0.5–1.0)	1.0 (0.8–1.4)	1.2 (0.9–1.6)	0.035
	**Long-term follow-up (n = 1808)**				
	**Quartile of SD of diastolic blood pressure, range in mmHg**				
	**Group 1 (n = 431) ≤5.5**	**Group 2 (n = 452) >5.5−≤7**	**Group 3 (n = 460) >7−≤8.8**	**Group 4 (n = 465) >8.8**	**P for heterogeneity**
Coronary events (n = 248)	1 (ref)	1.3 (0.9–1.9)	1.1 (0.7–1.6)	1.8 (1.3–2.6)	0.002
Fatal/non-fatal stroke (n = 245)	1 (ref)	1.1 (0.7–1.5)	1.0 (0.7–1.4)	1.3 (0.9–1.8)	0.470
Heart failure hospitalisation (n = 216)	1 (ref)	1.7 (1.1–2.5)	1.2 (0.8–1.8)	1.9 (1.3–2.8)	0.005
Vascular mortality (n = 315)	1 (ref)	1.3 (0.9–1.8)	1.1 (0.8–1.5)	1.6 (1.2–2.2)	0.020
Total mortality (n = 735)	1 (ref)	1.2 (0.9–1.5)	1.1 (0.9–1.3)	1.4 (1.1–1.7)	0.012

Data are presented as Hazard Ratios and 95% Confidence Intervals.

Adjustment for randomized treatment, country (short-term follow-up only), mean diastolic blood pressure, age, gender, current smoker, histories of diabetes, hypertension, cardiovascular disease, cerebrovascular disease & peripheral vascular disease, body mass index, high-density lipoprotein and low-density lipoprotein.

### Statistical Analysis

Baseline summary characteristics are reported as means with standard deviations (SD) for continuous variables and as numbers with percentage (%) for categorical variables. Variability of blood pressure was quantified using the standard deviation (SD) and the coefficient of variation (SD/mean; CV). The results for SD and CV were qualitatively the same; therefore the results for SD are presented. F-tests were used to test the difference in blood pressure variability between participants receiving pravastatin and those receiving placebo. The association of visit-to-visit variability in blood pressure in relation to the different endpoints was assessed separately for short- and long-term follow-up, the latter restricted to the Scottish sub-cohort. For short-term follow-up blood pressure variability was calculated from measurements made at visits 1 to 5 (0–12 months). In the Scottish sub-cohort which, in addition, has longer-term follow-up, blood pressure variability was calculated from measurements made from visit 1 to 9 (0–24 months). Participants who had a CVD event during the blood pressure variability measurement period (0–12 months for short-term follow-up and 0–24 months for long-term follow-up) were excluded from relevant analysis. Participants with one or more missing blood pressure measurements, including those who died during the blood pressure variability measurement period, were excluded from the analyses. The agreement in blood pressure variability was assessed for the short-term inception cohort by analysing the Spearman Rank Correlation between the first three blood pressure measurements and the last two measurements. For the long-term Scottish sub-cohort, agreement in blood pressure variability was assessed by analysing the Spearman Rank Correlation between the first five blood pressure measurements and the last four measurements.

**Table 4:** Hazard Ratio’s for the endpoints associated with quartiles of the standard deviation (SD) of pulse pressure.

	Short-term follow-up (n = 4819)				
	Quartile of SD of pulse pressure, range in mmHg				
Outcomes	Group 1 (n = 1158)≤8	Group 2 (n = 1149)>8− ≤11	Group 3(n = 1231)>11−≤15	Group 4(n = 1281) >15	P for heterogeneity
Coronary events (n = 407)	1 (ref)	0.8 (0.6–1.1)	0.9 (0.7–1.2)	0.8 (0.6–1.0)	0.287
Fatal/non-fatal stroke (n = 158)	1 (ref)	0.9 (0.5–1.4)	0.9 (0.6–1.4)	1.2 (0.8–1.8)	0.526
Heart failure hospitalisation (n = 144)	1 (ref)	1.3 (0.8–2.1)	1.0 (0.6–1.6)	1.1 (0.7–1.7)	0.575
Vascular mortality (n = 172)	1 (ref)	1.1 (0.7–1.7)	1.2 (0.8–1.8)	1.0 (0.6–1.5)	0.761
Total mortality (n = 330)	1 (ref)	1.3 (0.9–1.8)	1.3 (0.9–1.8)	1.2 (0.9–1.7)	0.415
	**Long-term follow-up (n = 1808)**				
	**Quartile of SD of pulse pressure, range in mmHg**				
	**Group 1 (n = 459) ≤9.5**	**Group 2 (n = 422)** **>9.5−≤12**	**Group 3 (n = 475)** **>12−≤15**	**Group 4 (n = 452) >15**	**P for heterogeneity**
Coronary events (n = 248)	1 (ref)	0.9 (0.6–1.3)	1.0 (0.7–1.5)	1.1 (0.8–1.6)	0.727
Fatal/non-fatal stroke (n = 245)	1 (ref)	1.1 (0.7–1.6)	1.5 (1.0–2.1)	1.6 (1.1–2.4)	0.024
Heart failure hospitalisation (n = 216)	1 (ref)	0.8 (0.6–1.3)	1.1 (0.7–1.6)	1.2 (0.8–1.7)	0.357
Vascular mortality (n = 315)	1 (ref)	0.8 (0.6–1.2)	1.1 (0.8–1.6)	1.3 (1.0–1.8)	0.031
Total mortality (n = 735)	1 (ref)	1.1 (0.9–1.3)	1.2 (1.0–1.5)	1.3 (1.1–1.6)	0.068

Data are presented as Hazard Ratios and 95% Confidence Intervals.

Adjustment for randomized treatment, country (short-term follow-up only), mean pulse pressure, age, gender, current smoker, histories of diabetes, hypertension, cardiovascular disease, cerebrovascular disease & peripheral vascular disease, body mass index, high-density lipoprotein and low-density lipoprotein.

The associations between measures of blood pressure variability and time to occurrence of clinical outcomes were assessed using Cox proportional hazards models. Measures of blood pressure variability used were standard deviations and these were split into quarters of their distributions and hazard ratios (HRs) and corresponding 95% confidence intervals were calculated in relation to the lowest quarter of SD (referent); homogeneity across the quartiles was assessed using a general test of heterogeneity.

**Table 5:** Hazard Ratio’s for the endpoints associated with one SD change in each blood pressure parameter (a–c) for the long-term follow-up (n = 1808).

a. Systolic blood pressure.						
	Change of one standard deviation					
	Baseline SBP (SD = 20.80 mmHg)		Mean SBP (SD = 15.53 mmHg)		SD SBP (SD = 4.88 mmHg)	
Outcomes	HR (95% CI)	p-value	HR (95% CI)	p-value	HR (95% CI)	p-value
Coronary events	1.1 (0.9–1.2)	0.390	1.2 (1.0–1.32)	0.030	1.1 (1.0–1.3)	0.044
Fatal/non-fatal stroke	1.2 (1.0–1.3)	0.030	1.2 (1.1–1.4)	0.003	1.1 (1.0–1.3)	0.084
Heart failure hospitalisation	1.1 (0.9–1.2)	0.514	1.1 (0.9–1.2)	0.426	1.1 (1.0–1.3)	0.135
Vascular mortality	1.1 (1.0–1.2)	0.082	1.2 (1.1–1.3)	0.005	1.2 (1.1–1.4)	>0.001
Total mortality	1.0 (1.0–1.1)	0.255	1.1 (1.0–1.2)	0.056	1.1 (1.1–1.2)	>0.001
**b. Diastolic blood pressure**						
	**Change of one standard deviation**					
	**Baseline DBP (SD = 10.80 mmHg)**		**Mean DBP (SD = 7.13 mmHg)**		**SD DBP (SD = 3.66 mmHg)**	
**Outcomes**	**HR (95% CI)**	**p-value**	**HR (95% CI)**	**p-value**	**HR (95% CI)**	**p-value**
Coronary events	1.0 (0.9–1.1)	0.989	1.0 (0.9–1.1)	0.939	1.2 (1.1–1.4)	0.001
Fatal/non-fatal stroke	1.2 (1.0–1.3)	0.019	1.2 (1.1–1.4)	0.008	1.1 (1.0–1.2)	0.141
Heart failure hospitalisation	0.9 (0.8–1.0)	0.181	0.9 (0.8–1.0)	0.060	1.2 (1.0–1.3)	0.014
Vascular mortality	1.0 (0.9–1.2)	0.558	1.0 (1.0–1.2)	0.234	1.2 (1.1–1.3)	0.003
Total mortality	1.0 (1.0–1.1)	0.465	1.0 (1.0–1.1)	0.459	1.1 (1.1–1.2)	>0.001
**c. Pulse pressure**						
	**Change of one standard deviation**					
	**Baseline PP (SD = 17.70 mmHg)**		**Mean PP (SD = 12.38 mmHg)**		**SD PP (SD = 4.33 mmHg)**	
**Outcomes**	**HR (95% CI)**	**p-value**	**HR (95% CI)**	**p-value**	**HR (95% CI)**	**p-value**
Coronary events	1.1 (0.9–1.2)	0.314	1.2 (1.1–1.4)	0.006	1.1 (1.0–1.2)	0.199
Fatal/non-fatal stroke	1.1 (0.9–1.2)	0.284	1.2 (1.0–1.3)	0.031	1.2 (1.0–1.3)	0.018
Heart failure hospitalisation	1.1 (1.0–1.3)	0.118	1.2 (1.0–1.3)	0.043	1.1 (0.9–1.2)	0.271
Vascular mortality	1.1 (1.0–1.2)	0.094	1.2 (1.1–1.3)	0.005	1.2 (1.0–1.3)	0.008
Total mortality	1.0 (1.0–1.1)	0.375	1.1 (1.0–1.2)	0.053	1.1 (1.0–1.2)	0.008

Data are presented as Hazard Ratios and 95% Confidence Intervals.

Adjustment for randomized treatment, country (short-term follow-up only), mean pulse pressure, age, gender, current smoker, histories of diabetes, hypertension, cardiovascular disease, cerebrovascular disease & peripheral vascular disease, body mass index, high-density lipoprotein and low-density lipoprotein.

Analyses were adjusted for country (short-term analyses only), randomized treatment group (pravastatin or placebo) and the respective mean blood pressure measure during the period blood pressure variability was assessed (mean systolic blood pressure for systolic blood pressure variability; mean diastolic blood pressure for diastolic blood pressure variability and mean pulse pressure for pulse pressure variability) (Model 1). A second model (Model 2) included additional adjustment for age, gender, smoking status, and prior histories of diabetes, hypertension, cardiovascular disease, cerebrovascular disease or peripheral vascular disease, as well as body mass index (BMI), high density lipoprotein cholesterol (HDL) and low density lipoprotein cholesterol (LDL). The results for Models 1 and 2 were qualitatively the same; therefore the results for Model 2 are presented in the main tables.

We performed a number of sensitivity analyses, including using continuous values of the blood pressure variability measurements to evaluate the influence of the splitting the blood pressure variability measurements by quartiles. In this case continuous measures of variability of blood pressure were reported as HRs per 5 mmHg increase in SD of systolic and diastolic blood pressure and pulse pressure. HRs for one SD difference in baseline blood pressure, mean blood pressure and blood pressure variability, for systolic and diastolic blood pressure and pulse pressure were calculated. Further subgroup analyses were conducted for gender, the use of antihypertensive medication at baseline, baseline blood pressure above and below the median and for patients with/without a history of a stroke or transient ischaemic attack.

## Results

Of the initial cohort of 5804 PROSPER participants, 5054 were alive and had a full blood pressure profile up to 12 months (five measurements); 235 of these participants were excluded from analyses as having had a CVD event during this period, giving 4819 participants as an inception cohort to be included in the short-term (in trial) follow-up analyses (**Figure 1**). For the long-term follow-up (including post-trial) analyses only the Scottish sub-cohort was eligible (n = 2520); 625 of these participants were excluded because they did not have a full blood pressure profile (up to two years, nine measurements); an additional 87 of these participants were excluded as they had a CVD event during this period, giving 1808 Scottish participants to be included in the inception cohort for the long-term follow-up analyses (**Figure 1**).

**Table 1** presents the baseline characteristics for the participants in the short- and long-term follow-up. Of the 4819 participants in the short-term follow-up 2339 (48.5%) were men, the mean age was 75.2 years (SD 3.3) and 2086 (43.3%) had a history of cardiovascular disease. Of the 1808 participants in the long-term follow-up 876 (48.5%) were men, the mean age was 75.2 years (SD 3.4) and 865 (47.8%) had a history of cardiovascular disease.

We initially examined whether there was a difference in variability in blood pressure between participants receiving pravastatin and those receiving placebo. There was no significant difference for short-term and long-term follow up, systolic and diastolic blood pressure and pulse pressure (range of p-values 0.288–0.868); therefore, data from both groups were combined; however all subsequent analyses were adjusted for randomized treatment group because of the effect of the pravastatin on CVD outcomes.

Blood pressure variability was reproducible for short-term and long-term follow up, for systolic and diastolic blood pressure and pulse pressure (p-value <0.0001). The Spearman Rank Correlation was higher when more blood pressure measurements were added in the model.

### Visit-to-visit Variability in Systolic Blood Pressure

Across the first five blood pressure measurements in the short-term follow-up cohort the mean SD for variability of systolic blood pressure was 13.6 mmHg. The mean SD for variability of systolic blood pressure across the first nine blood pressure measurements in the long-term follow-up cohort was 14.1 mmHg. **Table 2** shows the results of the time-to-event analyses for the different quartiles of SD of systolic blood pressure for all endpoints in the short-term and long-term follow-up. In the long-term follow-up, risk of vascular and total mortality increased across quartiles for SD of systolic blood pressure in the fully adjusted model. SD of systolic blood pressure per 5 unit change (mmHg) was associated with coronary events (HR 1.1 (1.0–1.3); vascular mortality (HR 1.2, 95% CI 1.1–1.4) and total mortality (HR 1.1, 95% CI 1.1–1.2) in the long-term follow-up. The predictive value of visit-to-visit variability in systolic blood pressure was similar in all subgroup analyses, including in participants with and without the use of antihypertensive medication (data not shown).

### Visit-to-visit Variability in Diastolic Blood Pressure

Across the first five measurements in the whole cohort the mean SD of diastolic blood pressure was 7.3 mmHg. The mean SD of diastolic blood pressure across the first nine measurements in the Scottish sub-cohort was 7.4 mmHg. **Table 3** shows the results of the time-to-event analyses for the different quartiles of diastolic blood pressure for all endpoints in the short-term and long-term follow-up. In both short-term and long-term follow-up, high visit-to-visit variability in diastolic blood pressure was associated with increased risk of coronary events, heart failure hospitalisation and vascular and total mortality. The HRs for heart failure hospitalisation and coronary events in the long-term follow-up were 1.9 (95% CI 1.3–2.8) for the highest quarter versus lowest quarter of SD of diastolic blood pressure and 1.8 (95% CI 1.3–2.6), respectively in the fully adjusted model (**Table 3**). SD of diastolic blood pressure per 5 unit change (mmHg) predicted coronary events (HR 1.1, 95% CI 1.0–1.3) and heart failure hospitalisation (HR 1.3, 95% CI 1.1–1.6) in the short-term follow-up. In the long-term follow-up SD of diastolic blood pressure per 5 unit change predicted coronary events (HR 1.5, 95% CI 1.2–1.8); heart failure hospitalisation (HR 1.4, 95% CI 1.1–1.8); vascular mortality (HR 1.4, 95% CI 1.1–1.7) and total mortality (HR 1.3, 95% CI 1.1–1.5).

Variability in diastolic blood pressure was more predictive for coronary events in male participants (p for interaction = 0.008) and for vascular mortality in male participants and participants with systolic blood pressure below median (p for interaction = 0.043 and 0.028, respectively) in long-term follow-up.

### Visit-to-visit Variability in Pulse Pressure

Across the first five measurements in the whole cohort the mean SD of pulse pressure was 12.2 mmHg. Across the first nine measurements in the Scottish sub-cohort the mean SD of pulse pressure was 12.6 mmHg.

**Table 4** shows the results of the time-to-event analyses for the different quartiles of pulse pressure for all endpoints in the short-term and long-term follow-up. In the short term follow-up, there was no association between the SD of pulse pressure and the risk of CVD events or mortality. In the long-term follow-up, high visit-to-visit variability in pulse pressure was associated with increased risk of stroke (HR for the highest quartile versus lowest quartile 1.6, 95% CI 1.1–2.4); vascular and total mortality (HR 1.3, 95% CI 1.0–1.8 and HR 1.3, 95% CI 1.1–1.6 respectively). When the analyses were repeated for the continuous values of pulse pressure, SD of pulse pressure per 5 unit change (mmHg) predicted stroke (HR 1.2, 95% CI 1.0–1.4), vascular mortality (HR 1.2, 95% CI 1.0–1.3) and total mortality (HR 1.1, 95% CI 1.0–1.2) in the long-term follow-up. Variability in pulse pressure was more predictive for total mortality in participants with systolic blood pressure below the median (p for interaction = 0.024).

### Sensitivity Analyses

Table 5 shows the result of the analyses with one SD difference in baseline, mean and variability in blood pressure in the long-term Scottish cohort. One SD difference in baseline systolic and diastolic blood pressure, but not pulse pressure, was associated with an increased risk of stroke. One SD difference in mean systolic and diastolic blood pressure and pulse pressure was associated with an increased risk of stroke.

One SD difference in variability in systolic and diastolic blood pressure predicted an increased risk of vascular and total mortality, but was not associated with an increased risk of stroke.

In the analyses with one SD difference in baseline, mean and variability in blood pressure in the short-term follow-up cohort, one SD difference in variability in diastolic blood pressure predicted an increased risk in coronary events and heart failure hospitalisation (HR 1.11, 95% CI 1.01–1.21 and 1.20, 95% CI 1.05–1.38, respectively), no other associations were found (data not shown).

## Discussion

This study shows that in older subjects visit-to-visit variability in systolic blood pressure, diastolic blood pressure and pulse pressure are associated with an increased long-term risk for cardiovascular and total mortality. In addition variability in diastolic blood pressure was predictive of coronary events and heart failure hospitalisation; variability in systolic blood pressure was predictive of heart failure hospitalisations. Variability in pulse pressure (but not diastolic blood pressure or systolic blood pressure) was somewhat associated with long-term stroke risk. These associations were independent of respective mean blood pressure values, the use of antihypertensive medication and other risk factors.

The association of intra-individual variability in blood pressure measurements with adverse clinical outcomes was first recognised in the early 1990s [18]–[21]. Subsequent studies have investigated the predictive value of visit-to-visit variability in blood pressure in middle-aged people [19], [22]–[25]. In contrast the present study population consists of an older population (aged ≥70 years) with high risk of vascular events and deaths. Therefore, the present study gives new insight into the clinical significance of blood pressure variability with regard to morbidity and mortality in later life. It has been suggested that the association between increased variability in blood pressure and the risk of stroke is strongest in younger age groups [10], [13].

The reliability of blood pressure variability increased with the number of measurements included, this is in line with previous research in younger age groups [10], [26]. It is possible that our measures of blood pressure variability, although based on a reasonably large number of individual measurements, may still have underestimated the true magnitude of effect of variability on clinical outcomes.

The association between visit-to-visit variability in systolic blood pressure and increased total mortality found in older persons in this present study is generally in line with previous research in younger age-groups [13], [14]. Visit-to-visit variability in systolic blood pressure has also been claimed to be a predictor of stroke [10], [13], [24], [27] and coronary events [13], [28], however in the present study visit-to-visit variability in systolic blood pressure was not associated with an increased risk of stroke or coronary events. In contrast, sensitivity analyses in this present study showed that one SD difference in baseline and mean systolic and diastolic blood pressure was associated with an increased risk of stroke.

In our cohort, variability in pulse pressure was the only measure that was associated with an increased risk of stroke, albeit modestly so; systolic blood pressure variability may be a more powerful predictor of stroke in younger cohorts; Rothwell *et al.* [13] found an adjusted HR for stroke of 12.1 (95% CI 7.4–19.7; highest vs. lowest decile) in a population aged 40–79 years. Such differences between our study and other cohorts might be caused by higher mean systolic blood pressure levels in our older cohort. However there was no meaningful difference in average blood pressure between our study and other relevant cohorts (e.g. mean systolic blood pressure 150 mmHg in the UK-TIA and 164 mmHg at baseline and 148 mmHg on treatment in the ASCOT-BPA [13] compared to 154/155 mmHg in the present study).

In previous research, in predominantly younger populations compared to the present study, no associations were present between visit-to-visit variability in diastolic blood pressure and all-cause mortality and CVD [13], [14], [28]. In contrast, our data suggest that in older subjects diastolic blood pressure variability is more strongly associated with coronary events and vascular or total mortality than is systolic pressure variability, especially in male subjects and those with systolic blood pressure below median. The mechanisms for why diastolic blood pressure variability should be more strongly associated with risk in elderly remain uncertain but could speculatively include a bigger drop off in diastolic blood pressure as marker of risk. In addition, it is also not clear why such associations appear to be significantly stronger in men compared to women. Clearly, our results suggest these issues merit further study.

The present study reveals some differences in the association between variability in the different blood pressure measurement and outcomes and shows some differences with previous research. One of the major differences between the present study and previous research is the age of the participants. Although the present study was not aimed to investigate etiological mechanisms behind the observed associations, it is tempting to hypothesize that the mechanisms involved in the association between variability in blood pressure and stroke are different in younger and older persons. In previous studies, greater variability in blood pressure was related to older age and pulse pressure [13], [14], which both correlate with arterial stiffness. Arterial stiffness may play a role in the found associations between variability in blood pressure and CVD events. While variability increases with age, the association with CVD events is not found to increase correspondingly with similar analysis. The variability in systolic blood pressure found in the present study was indeed higher than the variability in systolic blood pressure found in younger populations (mean SD of systolic blood pressure 14.1 mmHg vs. 7.7 mmHg [14]), while the associated risk for total mortality was not higher. This could indicate that there might be more competing mechanisms in older persons than in younger persons.

This study has a number of strengths. Blood pressure was not part of the inclusion or exclusion criteria for the PROSPER trial. Therefore, people with the full range of baseline blood pressure and variability in blood pressure were included. The estimation of visit-to-visit variability should be reasonably reliable because of the frequency of measurements. The large sample size allowed us to conduct several subgroup analyses and investigating different outcomes. However a limitation of this study is that long-term follow-up was not assessed in the total PROSPER trial population and it was only available for the Scottish sub-cohort. Another potential limitation is that the participants were randomized to an intervention (pravastatin vs. placebo), however, we found no difference in variability in blood pressure between randomized groups and all analyses were adjusted for the randomized treatment. Third, we had no data on the use of antihypertensive medications during the follow-up after the randomized control trial ended. PROSPER was not designed to assess the effect of blood pressure on outcomes and the accuracy of measurement of blood pressure was reduced. By not having the perfect blood pressure measurement we may have underestimated the true effect of variability in blood pressure on clinical outcomes.

The present study aimed only to establish whether there is an association between of visit-to-visit variability in blood pressure and adverse CVD outcomes in older patients; however if these associations are causal the results may have implications in the management and treatment of blood pressure in the older population. Besides the aim of lowering the usual level of blood pressure in hypertensive patients, it is possible that additional benefit might be obtained from reducing variability in blood pressure. In a recent meta-analysis it was suggested that the use of calcium channel blockers and non-loop diuretics results in less systolic blood pressure variability than the use of ACE inhibitors and angiotensin 2 receptor antagonists. Calcium channel blockers have shown to reduce visit-to-visit variability compared with placebo [29]. However, currently it is not certain whether differential effects of various antihypertensives on variability in blood pressure will also lead to clinical gains.

In conclusion, in older subjects at risk of CVD events variability in systolic blood pressure is predictive for the risk of heart failure hospitalisations and cardiovascular and total mortality; variability in diastolic blood pressure is predictive for the risk of coronary events, and vascular and total mortality; variability in pulse pressure is predictive of stroke.
